# Long-term outcome of cutaneous melanoma patients treated with boron neutron capture therapy (BNCT)

**DOI:** 10.1093/jrr/rraa068

**Published:** 2020-09-29

**Authors:** Junichi Hiratsuka, Nobuhiko Kamitani, Ryo Tanaka, Ryoji Tokiya, Eisaku Yoden, Yosinori Sakurai, Minoru Suzuki

**Affiliations:** Department of Radiation Oncology, Kawasaki Medical School, 577 Matsushima, Kurashiki City, Okayama 701-0192, Japan; Department of Radiation Oncology, Kawasaki Medical School, 577 Matsushima, Kurashiki City, Okayama 701-0192, Japan; Department of Dermatology, Kawasaki Medical School, 577 Matsushima, Kurashiki City, Okayama 701-0192, Japan; Department of Radiation Oncology, Kawasaki Medical School, 577 Matsushima, Kurashiki City, Okayama 701-0192, Japan; Department of Radiation Oncology, Kawasaki Medical School, 577 Matsushima, Kurashiki City, Okayama 701-0192, Japan; Department of Particle Radiation Oncology, Integrated Radiation and Nuclear Science, Kyoto University, Kumatori, Osaka 590-0494, Japan; Department of Particle Radiation Oncology, Integrated Radiation and Nuclear Science, Kyoto University, Kumatori, Osaka 590-0494, Japan

**Keywords:** BNCT, celanoma, ckin, clinical results

## Abstract

Our aim was to assess the long-term clinical outcome of boron neutron capture therapy (BNCT) using ^10^B-para-boronophenylalanine (BPA) as the boron delivery agent for cutaneous melanoma. Eight patients (eight lesions) were treated between October 2003 and April 2014. Their ages ranged from 48 to 86 years at the time of treatment. All of the targets were primary lesions and they were located on the sole or face. No patient had evidence of regional lymph node involvement, distant metastases or an active secondary cancer. The clinical stage was cT1-2N0M0 and performance scores were <2. BNCT was carried out at the Kyoto University Research Reactor (KUR). The patients were irradiated with an epithermal neutron beam between the curative tumor dose and the tolerable skin dose. Eight patients were evaluated and six showed a complete response (CR), while two patients had a partial response (PR). Of the two patients with a PR, one has remained a PR with brown spots persisting for 7.5 years following BNCT. The tumor in the other patient recurred after 6 years at the site of persisting brown macula. The overall control rate (CR + PR without recurrence) for the cohort was 88% (7/8). There have never been any adverse events >Grade 2 for the long follow-up period. Our results suggest that BNCT may be a promising treatment modality in the management of early stage cutaneous melanoma when wide local excision is not feasible.

## INTRODUCTION

Melanomas are malignant tumors arising from melanocytes, which are pigmented cells. Melanoma that occurs on the skin, known as cutaneous melanoma, is the most common type. The recommended treatment for cutaneous melanoma is wide surgical excision of the lesion with or without lymph node dissection, reconstruction with a skin graft or skin flap, and more recently, treatment with neoadjuvant chemoimmunotherapy [1, 2]. Although wide surgical excision remains the standard procedure, it is a highly invasive procedure, especially in older patients, and it may lead to a variety of functional, esthetic and psychological problems that result in a poor quality of life (QOL). Alternative treatment modalities, such as topical chemotherapy, immunotherapy and carbon ion or proton radiotherapy have been administered for local control when wide local excision is not feasible [3, 4].

In 1973, an experimental study of boron neutron capture therapy (BNCT) for malignant melanoma was initiated by Mishima and colleagues (Kobe University group). They first proposed employing BNCT for melanomas utilizing the specific melanin synthesis activity of melanoma cells. For this purpose, they first proposed employing BNCT using ^10^B-chlorpromazine as a boron carrier [5]. Subsequently, ^10^B-para-boronophenylalanine (BPA) was re-evaluated by the group [6]. BPA is selectively absorbed by melanoma cells and exhibits a high tumor to normal skin boron concentration ratio. After 15 years of basic research, this group initiated the first clinical study in 1987 for BNCT of cutaneous melanoma using BPA [7–13]. They also reported the radiation dose and local response of melanoma cases treated with BNCT between 1987 and 2001 [14]. Several groups [15–20] around the world have initiated the BNCT procedure following its development in the Japanese clinical study. Each group developed its own protocol and primary end point, however, the number of melanoma patients treated with BNCT has been too small. This is because surgical excision is considered to be the most effective and curative therapy for melanoma, which is known for having a high tolerance to irradiation. For the above reasons, there has been no literature regarding the long-term local response and survival of melanoma patients following BNCT. Using the research reactor, we initiated melanoma BNCT using the newly administered schedule of BPA and an epithermal neutron beam, beginning in 2003. We report the long-term clinical outcome of eight patients with cutaneous melanomas treated by BNCT alone. Although the patients in this study had received BNCT as an alternative therapy and as a therapeutic application for specific cases, and not as a prospective clinical trial, these results represent the first report that describes the long-term outcome of cutaneous melanoma patients treated with BNCT.

## MATERIALS AND METHODS

### Patients

Eight patients (eight lesions) with cutaneous melanoma were treated with BNCT between October 2003 and April 2014. Patient information and tumor characteristics are summarized in Table 1. Their ages ranged from 48 to 86 years at the time of treatment. All of the targets were primary lesions and they were located on the sole or face. Their diagnoses were confirmed histologically. Six patients were acral lentiginous melanoma (ALM) and two were lentigo maligna melanoma (LMM). The tumors and pigment plaque were measured by computed tomography (CT) scan, MRI, visual inspection or palpation immediately prior to BNCT. No patient had evidence of regional lymph node involvement, distant metastases or an active secondary cancer. The clinical stage was cT1-2N0M0 and performance scores were <2. All patients had been referred to our institution to receive BNCT as an alternative therapy and as a therapeutic application for specific cases, because they had refused to consent to surgical excision or they were inoperable due to complications. Thus, BNCT was first-line therapy for these patients. Patients gave informed consent to undergo BNCT and approval for the study was obtained from the Kawasaki Medical School and the Kyoto University Medical and Ethics Committee.

**Table 1:** Patient information and tumor characteristics

Patient No.	Age, years/gender^a^	WHO PS^b^	Site of primary tumor and subtype of histology	Clinical stage	Largest black macula (cm)
1	72/M^b^	2	Sole ALM	T1aN0M0	2.5
2	85/F	1	Sole ALM	T2aN0M0	2.5
3	85/F	2	Sole ALM	T2aN0M0	4.2
4	71/F	1	Sole ALM	T1aN0M0	2.8
5	81/F	1	Sole ALM	T1aN0M0	5.5
6	48/F	1	Sole ALM	T1aN0M0	5.5
7	86/F	1	Face LMM	T1aN0M0	8.3
8	84/F	1	Face LMM	T1aN0M0	3.5

^a^M = male, F = female.

^b^PS = performance status.

### BNCT

**Fig. 1.**
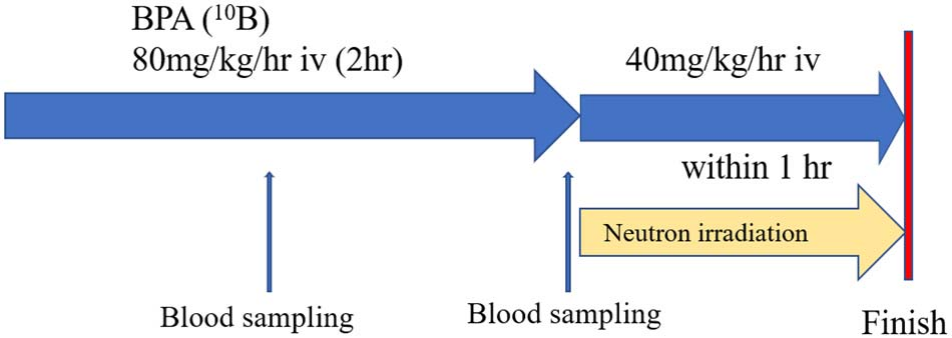
Patients were administered BPA-fructose complex (BPA-F) at 80 mg/kg/h. intravenously (iv) for 2 h. After the start of first administration of BPA, whole blood sampling was performed at 1 and 2 h. Following blood sampling at 2 h, neutron irradiation was started simultaneously with the start of the second administration of BPA at 40 mg/kg/h. Neutron irradiation was carried out during the last hour during infusion of BPA-F. At the same time as finishing irradiation, continuous infusion of BPA was ended.

The BNCT protocol used in this study was based on the treatment procedure developed by Mishima *et al*. [11, 14]. BNCT was carried out at the Kyoto University Research Reactor (KUR) operating at 5 MW of power using an epithermal neutron beam. For all patients, a 10 mm thick plate, composed of human body equivalent material, was placed over the area to be irradiated in order to increase the thermalized neutron dose delivered to these superficial tumors. ^10^B-Enriched L-BPA, purchased from Interpharma Praha (Prague, Czech Republic), was used as the boron delivery agent. All patients were administered with BPA–fructose complex (BPA-F), which is more soluble in water than hydrochloride [8], at 80 mg/kg/h intravenously for 2 h. After the start of first administration of BPA, whole blood sampling was performed at 1 and 2 h, and ^10^B concentration was measured by prompt gamma-ray analysis [21]. Following blood sampling at 2 h, neutron irradiation was started simultaneously with the start of the second administration of BPA at 40 mg/kg/h. Neutron irradiation was carried out during the last hour during infusion of BPA-F. At the same time as finishing irradiation, continuous infusion of BPA was ended (Fig. 1).

Venous blood was drawn just before neutron beam irradiation. Fukuda *et al*. reported melanoma-to-blood and skin-to-blood boron concentration ratios obtained from patients who underwent surgical operations or skin biopsies in 1999 [22]. We applied these values. Boron concentration in melanoma and normal skin were estimated from the blood data and multiplied by factors of 3.0 and1.3, respectively [22].

Gold wires and small thermoluminescence detectors (TLD) of magnesium ortho-silicate (Mg_2_SiO_4_) were used for the measurement of neutron flux and γ-ray dose, respectively. These were attached to the skin at the site of the radiation field for dosimetry. Lithium fluoride (LiF) sheets (10 mm thick) were chosen as the collimator to shield normal tissues from neutron irradiation. The radiation field included a 2–3 cm safety margin surrounding the visible lesions. We provided no difference in the margin size between histological subtypes. All patients received BNCT without anesthesia.

### Dosimetry and absorbed dose calculation

BNCT consists of mixed radiation fields that differ in their linear energy transfer (LET). The total radiation dose in Gy, delivered to any tissue, can be expressed in Gy-equivalent (Gy-eq) units as the sum of each of the high LET dose components multiplied by the relative biological effectiveness (RBE), and more specifically, the compound biological effectiveness (CBE) factors [23, 24]. All absorbed doses were expressed in Gy-eq units using these factors.

RBE and CBE factors [24–26] used for converting the physical dose (Gy) to Gy-eq used in the BNCT clinical trial in Japan are shown in Table 2. The time for neutron irradiation ranged from 30 to 60 min, depending on the blood boron levels, tumor depth and the limiting dose to the nearest critical organ. In order to keep side effects at a minimum, neutron doses were restricted to not exceed the maximum tolerable dose of the surrounding normal tissue.

**Table 2:** RBE and CBE factors used for conversion of physical dose (Gy) into photon-equivalent dose (Gy-eq)

BNCT dose components	Tumor	Skin
^10^B (n, α)^7^Li	3.8	2.5
^14^N (n,p)^14^C	3.0	3.0
Ganma-ray	1.0	1.0

**Fig. 2.**
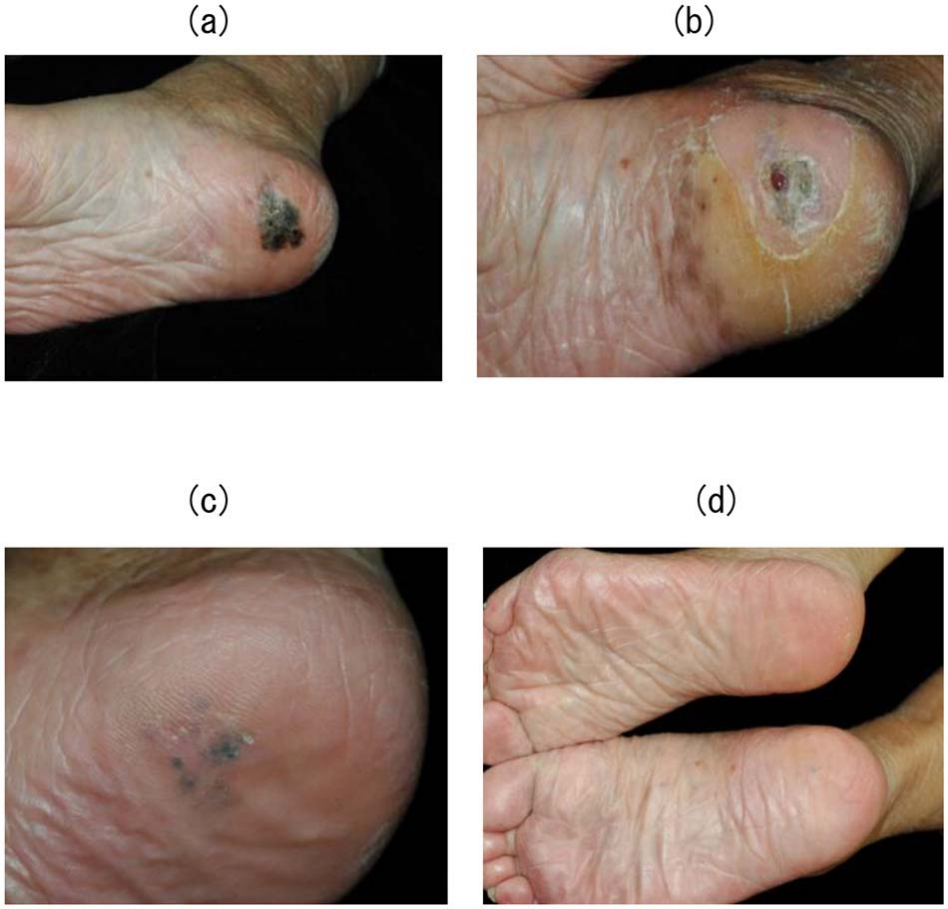
Case 3. (**a**) The appearance of the right sole with melanoma prior to BNCT (cT2aN0M0). (**b**) The tumor was reduced and dry erosion of the surrounding normal skin appeared a month after BNCT. (**c**) The skin reaction improved 3 months after BNCT. (**d**) Black macula disappeared completely after 9 months. The patient was pain-free when walking. This complete response continued for 5.5 years until her death at the age of 90 years.

The minimum dose for melanoma control in a single treatment was estimated to be 25 Gy-eq. The maximum tolerable doses to the skin in a single treatment were estimated to be 15 Gy-eq. A radiation dose that was less than the maximum tolerable dose and greater than the curative dose was selected. The Monte Carlo software package, Simulation Environment for Radiotherapy Applications (SERA), was used for dose planning [27].

### Evaluation of local response and survival

Tumor response was graded as follows: a complete response (CR) was considered as complete disappearance and regression of pigmented plaque and tumor by visual inspection, CT scan or MRI, while a partial response (PR) was designated if >50% disappearance and regression of plaque and tumor occurred. Initial tumor response was determined as the maximum reaction within 1 year following BNCT. Complication of normal skin and pain was graded according to the Common Terminology Criteria for Adverse Events (CTCAE) v.4.0. We evaluated the tumor response every 3 months in the first year, and thereafter every 6 months. Acute skin responses included those arising within the first 3 months after BNCT. Late responses were defined as those developing >3 months after the treatment. A survival analysis was made in January 2020. The survival time was calculated from the time of BNCT.

## RESULTS

### Local control and survival

**Fig. 3.**
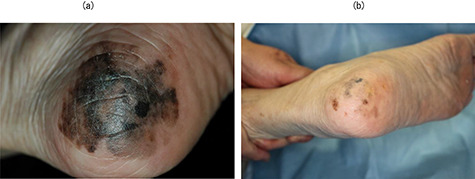
Case 5. (**a**) The appearance of the left sole with melanoma prior to BNCT (cT1aN0M0). The lesion contained a mixed black/brown macula. (**b**) Black macula in the lesion had completely disappeared; however, brown spots persisted for a long time period (7.5 years).

**Fig. 4.**
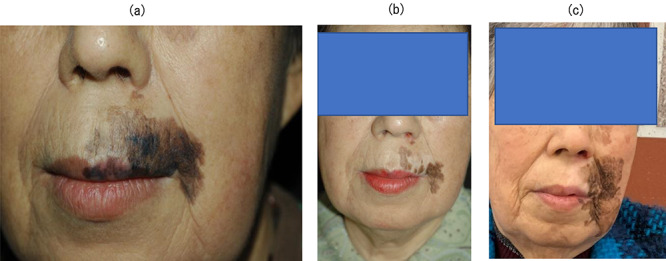
Case 7. (**a**) The appearance of the face with melanoma prior to BNCT (cT1aN0M0). The lesion contained a mixed black/brown macula. (**b**) The appearance of the face 1 year after BNCT. Black macula in the lesion had completely disappeared; however, brown spots persisted for a long time. This brown macula was evident for 6 years. (**c**) The appearance of the face 7 years after BNCT. The patient exhibited tumor recurrence after a long-term (6-year) persistence of brown macula.

All patients were followed for at least 5.6 years after BNCT or until death. No patients had been lost prior to follow-up. All patients showed similar responses in melanoma and normal skin after BNCT. All lesions gradually regressed along with depigmentation within a year. Of the eight patients, six showed CR. Fig. 2 shows the tumor response of case 3 with CR. Fig 2a shows the appearance of the right sole with melanoma prior to BNCT (cT2aN0M0). The tumor was reduced and dry erosion of the surrounding normal skin appeared a month after BNCT (Fig 2b). The skin reaction improved 3 months after BNCT (Fig 2c). Black macula disappeared completely after 9 months. The patient was pain-free when walking. This complete response continued for 5.5 years until her death at the age of 90 years (Fig 2d). Two out of eight patients exhibited PR. Of the two patients with a PR, one patient (case 5) has maintained PR status with persistence of brown spots for 7.5 years following BNCT. Fig. 3 shows the tumor response of case 5. Fig 3a shows the appearance of the left sole with melanoma prior to BNCT (cT1aN0M0). The lesion contained a mixed black/brown macula. After BNCT, black macula in the lesion had completely disappeared, however, brown spots persisted for 7.5 years (Fig 3b). In this case, there may be a strong possibility of clinical CR. The other patient (case 7) had tumor relapse after 6 years at the site of persisting brown macula. Fig 4 shows the tumor response of case 7. She developed facial melanoma (cT1aN0M0). The lesion contained a mixed black/brown macula (Fig 4a). Black macula in the lesion had completely disappeared, however, brown spots persisted for a long time after BNCT (Fig 4 b). She exhibited tumor relapse after 6 years at the site of persisting brown macula (Fig 4 c).

As shown in Figs 2–4, black macula in the lesion had completely disappeared. On the other hand, brown macula and spots have persisted for a long time period. There have been no local recurrences in the radiation field during follow-up except in case 7. The overall control rate (CR + PR without recurrence) for the cohort was 88% (7/8). Three patients died of pneumonia or advanced age with no local recurrence, whereas the remaining five patients are alive with good QOL and activities of daily living (ADL). The clinical outcome for each patient is shown in Table 3.

**Table 3:** Treatment administered and clinical outcome

Patient No.	Minimum tumor dose (Gy-eq)	Maximum skin dose (Gy-eq)	Adverse reaction	Tumor response	Locoregional control (years)	Survival
1	37	14	Grade 2	CR	12.6	12.6 years (death due to pneumonitis)
2	25	14	Grade 2	CR	6.9	6.9 years (death due to advanced age)
3	25	12	Grade 2	CR	5.5	5.5 years (death due to advanced age)
4	48	15	Grade 1	CR	8.2	8.2 years (alive with NED^a^)
5	50	13.5	Grade 2	PR	7.5	7.5 years (alive with PR)
6	41	12	Grade 2	CR	7.5	7.5 years (alive with NED)
7	33	8	Grade 1	PR	6.0	7.1 years (alive with recurrence in 6 years after NCT)
8	25	8	Grade 1	CR	5.6	5.6 years (alive with NED)

^a^NED = no evidence of disease.

### Complications

Acute responses generally consisted of edema, pigmentation and erosion and required no pain killer. All of these effects subsequently improved within several months. Late responses, such as ulceration or necrosis did not occur during the long follow-up period. There have never been any adverse events >Grade 2 (Table 3).

## DISCUSSION

In this study, we assessed the long-term clinical outcome of BNCT for cutaneous melanoma. Of the eight, six patients showed CR and two patients had PR in initial tumor response. There have been no local recurrences in the radiation field during follow-up except for one patient. The overall control rate (CR + PR without recurrence) for the cohort was 88% (7/8). There have not been any early and late complications that affected patient QOL or ADL. The clinical stage of all patients was cT1-2 N0, and six of them were T1aN0. In these cases, surgical excision also led to good results. Although wide surgical excision remains the standard procedure for cutaneous melanoma, it is a highly invasive procedure, especially in older patients, and it may lead to a variety of functional, esthetic and psychological problems that result in a poor QOL. On the other hand, BNCT is based on a nuclear reaction between the non-radioactive isotope ^10^B and thermal neutrons, and the resulting ^10^B (n, α)^7^Li capture reaction can eradicate cancer cells and spare surrounding normal cells. Theoretically, BNCT is an ideal type of radiation therapy since it is biologically rather than physically targeted and the structure and function of the normal tissues are spared. To date, the efficacy and safety of cutaneous melanoma BNCT has not been evaluated in a randomized clinical trial. The few case reports available indicate that BNCT may be effective in the treatment of cutaneous melanoma [15–20]. Although our patients had received BNCT alone as an alternative therapy, these results are the first to report on the long-term outcome of BNCT for cutaneous melanoma.

Melanoma has been regarded as a radioresistant tumor, demonstrating poor regression after photon radiotherapy. Even the use of a high dose per fraction protocol may only improve local response up to a CR rate of 20–30% [28]. Lately, several institutions have reported clinical results using Cf-252 neutron brachytherapy [29] or proton/carbon-ion radiotherapy [4] for cutaneous or mucosal melanoma, which enables much higher radiation doses to be delivered to the target. Many authors have reported clinical results for proton/carbon-ion radiotherapy for mucosal melanoma [30–32]; however, there has not been a large-scale clinical trial for cutaneous melanoma. Umebayashi *et al*. [33] reported the preliminary results of proton beam radiotherapy for cutaneous melanoma. Five primary melanomas and three metastatic lymph nodes were irradiated using a proton beam with a total dose of ~100 Gy, fractionated into single doses of ~10 Gy. As a result, all of the macular lesions and a primary melanoma tumor lesion disappeared completely, while no severe radiation-related complications occurred. Comparing the results of these retrospective studies is difficult because of the differences in patient and tumor characteristics and in the follow-up period.

BNCT is similar to carbon-ion radiotherapy in that much higher LET radiation doses can be delivered to the target volume; however, BNCT has a significant advantage over carbon-ion radiotherapy. BNCT can be administered to relatively large areas, thereby allowing a wide margin, because BPA selectively accumulates in melanoma cells, which are then killed by the ^10^B (n, α)^7^Li capture reaction without significant damage to the surrounding normal tissue. BNCT can deposit a large dose gradient at the cellular level between the melanoma and surrounding normal skin. In contrast, the dose of carbon-ion radiotherapy, which has a Bragg peak, is uniformly delivered within the target volume. This advantage of BNCT is particularly useful for treating melanoma because histological involvement characteristically extends beyond the grossly visible lesion. Thus, to add a large safety margin to the visible area is an important factor to avoid local recurrence both following surgery and radiotherapy.

Two of eight patients showed a PR. One patient has kept PR as persistence of brown spots for 7.5 years after BNCT, the other developed tumor regrowth after persistence of brown macula for 6 years. As is clear from Figs 2–4, black macula in the lesion completely disappeared. On the other hand, brown macula persisted for a long period. There is specuation about three possible reasons for this phenomenon. First*,* the boron concentration within the pheomelanin producing melanoma (brown macula) is very low compared with that of eumelanin production in melanoma (black macula). Melanocytic cells can produce two types of pigment, pheomelanin or eumelanin. The pathway of melanogenesis bifurcates into these two different pathways from dopaquinone, and there are no melanin monomers in the pheomelanogenesis pathway.

Yoshino, *et al*. demonstrated that enhanced eumelanogenesis induces higher production of melanin monomers. Melanin monomers, which are key intermediates for melanin polymer formation, play a critical role in ^10^B-BPA accumulation, because BPA can form a chemical complex with them [34]. The boron concentration in the brown macula may be much lower than expected and the low boron concentration is considered to cause the poor response of brown macula.

Second, the difference in color between black and brown macula is associated with the melanin synthesis activity of melanoma cells. Thus, the black macula promotes higher melanogenesis compared with the brown. Tsuboi *et al*. [35] reported enhanced melanogenesis induced by tyrosinase gene-transfer, which may increase boron uptake and the killing effect of BNCT for amelanotic melanoma. They concluded that the melanin synthesis activity had a strong influence on BPA uptake into melanoma cells. Third*,* the brown macula consists of melanophages, which phagocytize melanin granules that are released from melanoma cells killed by BNCT. In other words, it is stain of the skin made by same mechanism as tattoo and the phenomenon may be a histological regression of melanoma.

Recent advances in immunotherapeutic approaches [2, 36, 37] to treat metastatic melanoma combined with BNCT of the primary tumor might represent a breakthrough in treating this malignancy, which has a high propensity to metastasize. Targeting the programmed cell-death-1 (PD-1) ligand with multiple anti-PD-1 monoclonal antibodies has been evaluated in Phase III trials, and this treatment has resulted in impressive clinical responses. Since BNCT spares normal cells, and more specifically immune effector cells, it may complement immunotherapeutic approaches to treat melanoma. Thus, local BNCT combined with systemic immunotherapy are mutually complementary and potentially synergistic, since BNCT spares immune effector cells at the site of the tumor.

## CONCLUSIONS

Although patients in the present study had received BNCT as an alternative therapy and as a therapeutic application for specific cases and not as a prospective clinical trial, these results are the first to describe the long-term outcome of BNCT for cutaneous melanoma, which resulted in excellent local tumor control. Our results suggest that BNCT may be a promising treatment modality in the management of the early stage of cutaneous melanoma when wide local excision is not feasible.

## References

[1] Staiano J-J, Wong L, Butler J et al. Flap reconstruction following gynaecological tumour resection for advanced and recurrent disease--a 12year experience. J Plast Reconstr Aesthet Surg 2009;62:346–51. 18784004 10.1016/j.bjps.2007.12.050

[2] Wolchok J-D, Chiarion-Sileni V, Gonzalez R et al. Overall survival with combined Nivolumab and Ipilimumab in advanced melanoma. N Engl J Med 2017;377:1345–56. 28889792 10.1056/NEJMoa1709684 PMC5706778

[3] Janco JM, Markovic S-N, Weaver A-L et al. Vulvar and vaginal melanoma: Case series and review of current management options including neoadjuvant chemotherapy. Gynecol Oncol 2013;129:533–7. 23480869 10.1016/j.ygyno.2013.02.028

[4] Karasawa K, Wakatsuki M, Kato S et al. Clinical trial of carbon ion radiotherapy for gynecological melanoma. J Radiat Res 2014;55:343–50. 24536019 10.1093/jrr/rrt120 PMC3951082

[5] Mishima Y. Neutron capture treatment of malignant melanoma using ^10^B-chlorpromazine compound. In: McGovern VJ, Russell P (eds). Pigment Cell, vol 1, Mechanisms in Pigmentation. Basel: S. Karger, 1973, 215–21.

[6] Yoshino K, Kakihana H, Okamoto M et al. Chemical behavior of dopaborate and ^10^B-p-boronophenylalanine. In: Hatanaka H (ed). Neutron Capture Therapy. Niigata. Japan: Nishimura, 1986, 55–60.

[7] Fukuda H, Kobayashi T, Matsuzawa T et al. RBE of a thermal neutron beam and the ^10^B(n, α)^7^Li reaction on cultured B-16 melanoma cells. Int J Radiat Biol 1987;51:167–75. 10.1080/09553008714550601 3492464

[8] Yoshino K, Suzuki K, Mori Y et al. Improvement of solubility of p-boronophenylalanine by complex formation with monosaccarides. Strahlenther Onkol 1989;165:127–9. 2928932

[9] Hiratsuka J, Kono M, Mishima Y. RBEs of thermal neutron capture therapy and ^10^B (n, α) ^7^Li reaction on melanoma-bearing hamsters. Pigment Cell Res 1989;2:352–5. 2798330 10.1111/j.1600-0749.1989.tb00219.x

[10] Mishima Y, Ichihashi M, Hatta S et al. New thermal neutron capture therapy for malignant melanoma: Melanogenesis-seeking 10B molecule-melanoma cell interaction from in vitro to first clinical trial. Pigment Cell Res 1989;2:226–34. 2678078 10.1111/j.1600-0749.1989.tb00196.x

[11] Mishima Y, Honda C, Ichihashi M et al. Treatment of malignant melanoma by single neutron capture treatment with melanoma-seeking ^10^B-compound. Lancet 1989;2:388–9. 2569577 10.1016/s0140-6736(89)90567-9

[12] Hiratsuka J, Fukuda H, Kobayashi T et al. The relative biological effectiveness of ^10^B-neutron capture therapy for early skin reaction in the hamster. Radiat Res 1991;128:186–91. 1947014

[13] Fukuda H, Hiratsuka J, Honda C et al. Boron neutron capture therapy of malignant melanoma using ^10^B-paraboronophenylalanine with special reference to evaluation of radiation dose and damage to the normal skin. Radiat Res 1994;138:435–42. 8184019

[14] Fukuda H, Hiratsuka J, Kobayashi T et al. Boron neutron capture therapy (BNCT) for malignant melanoma with special reference to absorbed doses to the normal skin and tumor. Australas Phys Eng Sci Med 2003;26:78–84. 10.1007/BF03178777 14626847

[15] Busse P-M, Zamenhof R, Madoc-Jones H et al. Clinical follow-up of patients with melanoma of the extremity treated in a phase I boron neutron capture therapy protocol. In: Larsson B, Crawford J, Weinreich R (eds). Advances in Neutron Capture Therapy, Volume I. Amsterdam: Elsevier, 1997, 60–4.

[16] Palmer M-R, Goorley J-T, Kiger W-SIII et al. Treatment planning and dosimetry for the Harvard-MIT PhaseII clinical trial of cranial neutron capture therapy. Int J Radiat Oncol Biol Phys 2002;53:1361–79. 12128139 10.1016/s0360-3016(02)02862-6

[17] Wittig A, Sauerwein W, Moss R et al. Early phase I study of BNCT in metastatic malignant melanoma using the boron carrier BPA (EORTC protocol 11011). In: Nakagawa Y, Kobayashi T, Fukuda H (eds). Proceedings of ICNCT-12 Advances in Neutron Capture Therapy 2006. 2006, 284–7.

[18] Gonzales S-J, Bonomi M-R, Santacruz G-A et al. First BNCT treatment of a skin melanoma in Argentina: Dosimetric analysis and clinical outcome. Appl Radiat Isot 2004;61:1101–5. 15308199 10.1016/j.apradiso.2004.05.060

[19] Yong Z, Song Z, Zhou Y et al. Boron neutron capture therapy for malignant melanoma: First clinical case report in China. Chin J Cancer Res 2016;28:634–40. 28174492 10.21147/j.issn.1000-9604.2016.06.10 PMC5242447

[20] Morita N, Hiratsuka J, Kuwabara C et al. Successful BNCT for patients with cutaneous and mucosal melanomas: Report of 4 cases. In: Nakagawa Y, Kobayashi T, Fukuda H (eds). Proceedings of ICNCT-12. 2006, 18–20.

[21] Kobayashi T, Kanda K. Microanalysis system of ppm-order 10b concentrations in tissue for neutron capture therapy by prompt gamma-ray spectrometry. Nucl Instrum Methods Phys Res A 1983;204:525–31.

[22] Fukuda H, Honda C, Wadabayashi N et al. Pharmacokinetics of 10B-p-boronophenylalanine in tumors, skin and blood of melanoma patients: A study of boron neutron capture therapy for malignant melanoma. Melanoma Res 1999;9:75–83. 10338337 10.1097/00008390-199902000-00010

[23] Morris GM, Coderre JA, Hopewell JW et al. Response of rat skin to boron neutron capture therapy with p-boronophenylalanine or borocaptate sodium. Radiother Oncol 1994;32:144–53. 7972908 10.1016/0167-8140(94)90101-5

[24] Suzuki M, Kato I, Aihara T et al. Boron neutron capture therapy outcomes for advanced or recurrent head and neck cancer. J Radiat Res 2014;55:146–53. 23955053 10.1093/jrr/rrt098 PMC3885131

[25] Coderre JA, Morris GM. Review; the radiation biology of boron neutron capture therapy. Radiat Res 1999;151:1–18. 9973079

[26] Hopewell JW, Morris GM, Schwint A et al. The radiobiological principles of boron neutron capture therapy; a critical review. Appl Radiat Isot 2011;69:1756–9. 21543233 10.1016/j.apradiso.2011.04.019

[27] Nigg D-W, Wemple C-A, Wessol D-E et al. Sera -- an advanced treatment planning system for neutron therapy and bnct. Trans Am Nucl Soc 1999;10:66–8.

[28] Slingluff C-L, Flaherty K, Posenberg S-A et al. Cutaneous Melanoma. In: Devita V.T, Lawrence T.S, Rosenberg S.A (eds). Cancer: Principles and Practice of Oncology. 9^th^ edition. Philadelphia: Lippincott Williams &Wilkins, 2011, 1643–1691.

[29] Vtyurin B-M, Medvedev V-S, Anikin V-A et al. Neutron brachytherapy in the treatment of melanoma. Int J Radiat Oncol Biol Phys 1994;28:703–9. 8113115 10.1016/0360-3016(94)90197-x

[30] Tsuji H, Kamada T. A review of update clinical results of carbon ion radiotherapy. Jpn J Clin Oncol 2012;42:670–85. 22798685 10.1093/jjco/hys104 PMC3405871

[31] Kato M, Demizu Y, Saitoh J et al. Multicenter study of carbon-ion radiation therapy for mucosal melanoma of the head and neck: Subanalysis of the Japan carbon-ion radiation oncology study group (J-CROS) study (1402HN). Int J Radiat Oncol Biol Phys 2017;97:1054–60. 28332989 10.1016/j.ijrobp.2016.12.028

[32] Zenda S, Akimoto T, Mizumoto M et al. Phase II study of proton beam therapy as a nonsurgical approach for mucosal melanoma of the nasal cavity or Para-nasal sinuses. Radiother Oncol 2016;118:267–71. 26547102 10.1016/j.radonc.2015.10.025

[33] Umebayashi S, Uyeno K, Tsujii H et al. Proton radiotherapy for malignant melanoma of the skin. Dermatology 1995;190:210–3. 7599383 10.1159/000246687

[34] Yoshino K, Mishima Y, Kimura M et al. Capture of p-boronophenylalanine in malignant melanoma cells by complex formation with melanin monomers, DOPA, DHI and DHICA. In: Larsson B, Crawford J, Weinreich R (eds). Advances in neutron capture therapy. Amsterdam: Elsevier, 1997, 234–8.

[35] Tsuboi T, Kondoh H, Hiratsuka J et al. Enhanced melanogenesis induced by tyrosinase gene-transfer increases boron-uptake and killing effect of boron neutron capture therapy for amelanotic melanoma. Pigment Cell Res 1998;11:275–82. 9877098 10.1111/j.1600-0749.1998.tb00736.x

[36] Achkar T, Tarhini A-A. The use of immunotherapy in the treatment of melanoma. J Hematol Oncol 2017;10:88. 28434398 10.1186/s13045-017-0458-3 PMC5402170

[37] Yu Z, Si L. Immunotherapy of patients with metastatic melanoma. Chin Clin Oncol 2017;6:20. 28482673 10.21037/cco.2017.04.01

